# Overview on the Diversity of Sounds Produced by Clownfishes (Pomacentridae): Importance of Acoustic Signals in Their Peculiar Way of Life

**DOI:** 10.1371/journal.pone.0049179

**Published:** 2012-11-07

**Authors:** Orphal Colleye, Eric Parmentier

**Affiliations:** Laboratory of Functional and Evolutionary Morphology, University of Liège, Liège, Belgium; University Zürich, Switzerland

## Abstract

**Background:**

Clownfishes (Pomacentridae) are brightly colored coral reef fishes well known for their mutualistic symbiosis with tropical sea anemones. These fishes live in social groups in which there is a size-based dominance hierarchy. In this structure where sex is socially controlled, agonistic interactions are numerous and serve to maintain size differences between individuals adjacent in rank. Clownfishes are also prolific callers whose sounds seem to play an important role in the social hierarchy. Here, we aim to review and to synthesize the diversity of sounds produced by clownfishes in order to emphasize the importance of acoustic signals in their way of life.

**Methodology/Principal Findings:**

Recording the different acoustic behaviors indicated that sounds are divided into two main categories: aggressive sounds produced in conjunction with threat postures (charge and chase), and submissive sounds always emitted when fish exhibited head shaking movements (i.e. a submissive posture). Both types of sounds showed size-related intraspecific variation in dominant frequency and pulse duration: smaller individuals produce higher frequency and shorter duration pulses than larger ones, and inversely. Consequently, these sonic features might be useful cues for individual recognition within the group. This observation is of significant importance due to the size-based hierarchy in clownfish group. On the other hand, no acoustic signal was associated with the different reproductive activities.

**Conclusions/Significance:**

Unlike other pomacentrids, sounds are not produced for mate attraction in clownfishes but to reach and to defend the competition for breeding status, which explains why constraints are not important enough for promoting call diversification in this group.

## Introduction

In teleost fishes, the ability to produce sounds was developed independently in distant phylogenetic taxa [1]. To date, more than 100 fish families include species with the ability to emit sounds [2], [3]. The majority of acoustic signals are used in different behavioral contexts such as aggressive behavior (territorial defense, predator/prey interactions, competitive feeding) or reproductive activities (mate identification and choice, courtship, synchronization of gamete release) [2], [4], [5]. The diversity of sounds produced by fishes is not as remarkable as in other taxa; most fishes show poor amplitude and frequency modulation in their sounds [6], [7], [8] and have relatively limited acoustic repertoires. Only few fish species emit more than one or two distinct sound types. However, the calling characteristics provide sufficient information for species identification and communication. For example, the rainbow cichlid *Herotilapia multispinosa* emits four distinct sound types (thumps, growls, whoofs and volley sounds) that would result from two different sound-producing mechanisms [9]. The Lusitanian toadfish *Halobatrachus didactylus* produces the boatwhistle advertisement call that is related to the breeding season and at least three other sounds during agonistic encounters: grunts, croaks and double croaks [10], [11].

Damselfishes (Pomacentridae) are one of the best-studied families for the use of acoustic communication during courtship and agonistic interactions, with some species such as *Dascyllus albisella* and *D. flavicaudus* showing a great diversity and complexity in their acoustic repertoire. They are known to produce pulsed sounds during numerous behaviors including signal jump, mating/visiting, chasing conspecifics and heterospecifics, fighting conspecifics and heterospecifics, and nest cleaning [12], [13]. All these sounds seem to be constructed on the basis of the same mechanism since they display the same type of sound spectrum and show few differences in terms of pulse duration. On the other hand, differences in the number of pulses and pulse period could be due to the fish physiology reflecting the behavior and its motivational state [13].

Clownfishes (Pomacentridae) live in social groups in which there is a size-based dominance hierarchy [14], [15]. Within each group, numerous agonistic interactions occur and they appear to play an important role by maintaining size differences between individuals adjacent in rank [14], [15]. Intraspecific encounters are common and sometimes rather severe in their intensity. Larger fishes chase smaller ones, which means that the smallest one is the recipient of numerous charges [15]. All clownfish species have evolved ritualized threat and submissive postures that presumably serve to circumvent physical injury during intraspecific quarreling [16]. For example, the “head shaking” is considered as a submissive state exhibited by fish in reaction to aggressive interactions [16], [17], [18]. This behavior consists in a lateral quivering of the body that begins at the head and continues posteriorly.

Clownfishes are known to produce aggressive sounds while displaying charge and chase towards another specimen during agonistic interactions [16], [18], [19]. More recently, Colleye et al. [20] conducted further studies on aggressive sounds in the skunk clownfish *Amphiprion akallopisos*; they highlighted a size-related intraspecific variation in dominant frequency and pulse duration: smaller individuals produce higher frequency and shorter duration pulses than larger ones. Surprisingly, the relationship between fish size and both dominant frequency and pulse duration is not only species-specific. These relationships are also spread out over the entire tribe of clownfishes by being found among 14 different species that are situated on exactly the same slope, which means the size of any *Amphiprion* can be predicted by both acoustic features [21].

Besides these aggressive sounds, Schneider [18] documented a second type of sound that was associated with “head shaking” and was emitted by fishes in conjunction with submissive posture. Later, Allen [16] reported the presence of head shaking movements associated with sound emission during agonistic interactions between group members. Unfortunately, the lack of detailed acoustic data and the small sample sizes of the behavioral observations require further investigations to differentiate these sounds from aggressive ones and to better understand the scope of these acoustic signals within a social group of clownfishes.

Additionally, it was reported that clownfishes might produce sounds during courtship. Courtship in clownfishes is generally stereotyped and ritualized, and is typically accompanied by different activities such as nest cleaning, courtship, spawning and nest care [16]. Basically, studies that describe the courtship sounds in clownfishes are limited in number. To date, sound production during reproductive period has been reported in three clownfish species (*A. ocellaris*, *A. frenatus*, *A. sandaracinos*) by Takemura [22]. However, these observations need to be carefully considered since, according to the author, the sounds were hardly heard and sometimes they do not seem to be directly related to spawning behavior [22]. Therefore, deeper attention must be paid to confirm the implication of acoustic signals during reproduction in this group.

The present study aims to review and to synthesize the diversity of sounds produced by clownfishes in order to emphasize the importance of acoustic signals in their way of life. The purpose of this study is 1) to record and to analyze sounds associated with head shaking in order to determine their role in the social structure of clownfishes; 2) to determine whether clownfishes use acoustic signals to synchronize one or several of their reproductive activities and 3) to make further analyses of some results previously obtained for the aggressive sounds (see [20]) with the aim of determining whether some acoustic features may contribute to individuality.

## Materials and Methods

Different species and different types of data were collected in fish tanks and in the field with the aim of covering all the behaviors that could be associated with sound production. Experimental and animal care protocols followed all relevant international guidelines and were approved by the ethics commission (no. 728) of the University of Liège.

### Agonistic sounds

Three groups being each composed of four individuals of *Amphiprion frenatus* (Standard Length, SL: 44–112 mm) were collected by scuba diving on the fringing reef around Nakijin village (26°40′N – 127°59′E; Okinawa, Japan) during May and June 2009. All fish were then brought back with their host (*Entacmaea quadricolor*) to Sesoko Station, Tropical Biosphere Research Center, University of the Ryukyus where they were transferred to a community tank (3.5×2.0×1.2 m) filled with running seawater at ambient temperature (28 to 30.5°C). All fish were kept under natural photoperiod and fed once daily with food pellets *ad libitum*. The social rank of each individual was attested using size differences. Basically, groups were composed of a breeding pair and two non-breeders (Table 1).

**Table 1:** Standard length (SL) and size order in the different groups of *Amphiprion frenatus*.

Size order	SL (mm)		
	Group 1	Group 2	Group 3
α(female)	105	110	112
β(male)	76	81	83
γ(non-breeder)	63	65	75
δ(non-breeder)	44	50	53

Recordings were made in a smaller glass tank (1.2×0.5×0.6 m) filled with running seawater maintained at 28°C by means of a GEX cooler system (type GXC-201×, Osaka, Japan) for having standardized conditions. For the sound recordings, all individuals of a group and their host were first placed in the tank for an acclimation time of 2 days. Twenty sessions (each lasting around 45 minutes) were recorded during which interactions between group members were observed and noted in order to identify the sound emitter. Only sounds associated with head shaking movements were taken into account in the analyses because the aim of this part was to give a concise physical description of this type of sounds in order to compare it with clownfish aggressive sounds (see [20], [21], [23]).

Sound recordings were made using a Brüel & Kjaer 8106 hydrophone (sensitivity: −173 dB re. 1 V/µPa) connected via a Nexus™ conditioning amplifier (type 2690) to a Tascam HD-P2 stereo audio recorder (recording bandwith: 20 Hz to 20 kHz±1.0 dB). Thi system has a flat frequency response over wide range between 7 Hz and 80 kHz. The hydrophone was placed just above the sea anemone (±5 cm).

Sounds were digitized at 44.1 kHz (16-bit resolution) and analysed with AviSoft-SAS Lab Pro 4.33 software (1024 point Hanning windowed fast Fourrier transform (FFT)). Recording in small tanks induces potential hazards because of reflections and tank resonance [24]. A relevant equation [24] was thus used to calculate the resonant frequency of the tank, and a low pass filter of 2.05 kHz was applied to all sound recordings. Temporal features were measured from the oscillograms whereas frequency parameters were obtained from power spectra (filter bandwidth 300 Hz, FFT size point 256, time overlap 96.87% and a flat top window). Generally speaking, the recorded sounds were composed of a series of sounds being multiple-pulsed. The following sonic features were measured: pulse duration in ms, pulse period in ms (the average peak to peak interval between consecutive pulse units in a series), number of pulses per sound, sound duration in ms, sound period in ms (the average peak to peak interval between consecutive sounds in a train), number of sounds per train and dominant frequency in Hz (frequency component with the most energy). Note that sounds produced simultaneously by several individuals were excluded from acoustic analyses.

### Reproductive sounds

Recordings were made both in aquarium and in the field.

In captivity, three species (*A. akyndinos*, *A. melanopus* and *A. ocellaris*) reared for several years at Oceanopolis Aquarium in Brest (France) were recorded during July 2008. One mating pair per species was studied. Each mating pair was maintained in separate glass tanks (0.70×0.45×0.50 m) filled with running seawater at ambient temperature (26°C). The different reproductive activities (nest preparation, courtship, spawning and eggs care) were observed and recorded. Each recording session lasted approximately 2 h, and four recording sessions with a 1-hour interval were carried out by day in order to cover the whole daytime from dawn to dusk. Behaviors of male and female were observed and noted. In addition, recordings in captivity were also made in Sesoko Station. One mating pair of *A. clarkii* was kept in a tank (1.2×0.5×0.6 m) filled with running seawater maintained at 28°C. Recordings were carried out during summer season 2009 (between May and July) because reproduction is limited to this period when seawater temperatures are warmer.

In both cases, sound recordings were made using the same Brüel & Kjaer 8106 hydrophone (see above for details on material characteristics), and sounds were analyzed according to the procedure previously described.

Field recordings were made on the fringing reef in front of Hizuchi beach (26°11′N – 127°16′E; Akajima, Kerama Islands, Japan) in August 2009. They were made using a SONY HDD video camera placed in a housing (HC3 series) coupled with an external hydrophone (High Tech. Inc.) with a flat response of 20 Hz to 20 kHz and a nominal calibration of −164 dB re. 1 V/µPa (Loggerhead Instruments Inc.). Recordings were made by placing the housing in front of the inhabited sea anemone (distance of between 50 cm and 1 m) that lived at a depth of between 5 m and 10 m. Each recording session lasted from 1 to 4 h.

Behaviors associated with sound production were described and sounds were extracted in .wav files using the AoA audio extractor setup freeware (version 1.2.5). Sounds were digitised at 44.1 kHz (16-bit resolution), low-pass filtered at 1 kHz and analysed using AviSoft-SAS Lab Pro 4.33 software (1024 point Hanning windowed fast Fourrier Transform (FFT)). Only sounds with a good signal to noise ratio were included in the analyses.

### Statistical analyses

Correlations analyses were used to examine changes in all acoustic features of submissive sounds across SL. The data used in these analyses were mean values of all recorded sounds for each individual. Two statistical analyses were then performed to test the influence of social rank on sonic features. First, a full ANCOVA was run to test differences between social ranks (β, γ, δ; see Table 1) for the sonic variables correlated with SL. In this test, sonic variables are considered as variates, SL as a covariate and social rank is the grouping factor. Secondly, sonic variables not correlated with SL, which failed the test for normal distribution (Shapiro-Wilk *W* Test), were analyzed using a non-parametric Kruskal-Wallis one-way ANOVA by ranks with subsequent Dunn's test for pair-wise comparisons to test differences between social ranks. All statistical analyses were carried out with Statistica 7.1. Results are presented as means ± standard deviation (S.D.). Significance level was determined at *p*<0.05.

Mean ± S.D. values were calculated for each acoustic feature of agonistic sounds for all individuals. Overall means, S.D. and range values were subsequently calculated using each individual mean value for each variable. In order to compare between-individuals with within-individuals variability for each acoustic feature, the within-individuals coefficient of variance (C.V._w_ = S.D./mean) was calculated and compared with the between-individuals coefficient of variation (C.V._b_). The C.V._b_ was obtained by dividing the overall S.D. by the respective overall mean. The ratio C.V._b_/C.V._w_ was then calculated to obtain a measure of relative between-individuals variability for each acoustic feature. When this ratio assumes values larger than one, it suggests that an acoustic feature could be used as a cue for individual recognition [25], [26], [27]. Differences between individuals for each acoustic variable were tested using a Kruskal-Wallis analysis due to the lack of homogeneity of variance. Note that this statistical test was run between individuals from a same group in the case of submissive sounds. In addition, this test was also run between the 14 individuals of the skunk clownfish *Amphiprion akallopisos* for which aggressive sounds were previously recorded (see [20]), in order to determine if some acoustic features may contribute to individuality.

## Results

### Agonistic sounds

**Figure 1:**
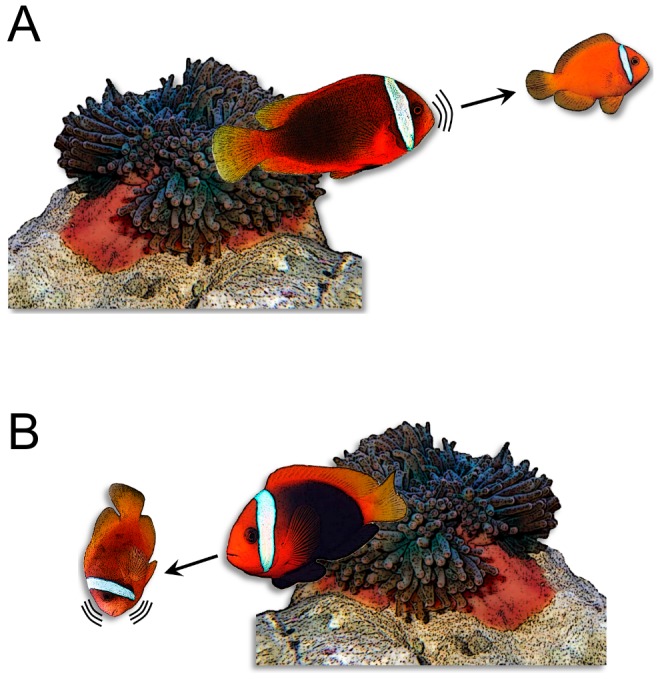
Behavioral postures associated with vocalizations and exhibited by *Amphiprion frenatus* during agonistic interactions. A) Dominant individual chasing subordinate while producing aggressive sounds. B) Head shaking movements displayed by subordinate while producing submissive sounds in reaction to aggressive act by dominant. Note that wiggly lines indicate the sound-producing individual, and arrows point out the receiver of the aggressive act.

**Figure 2:**
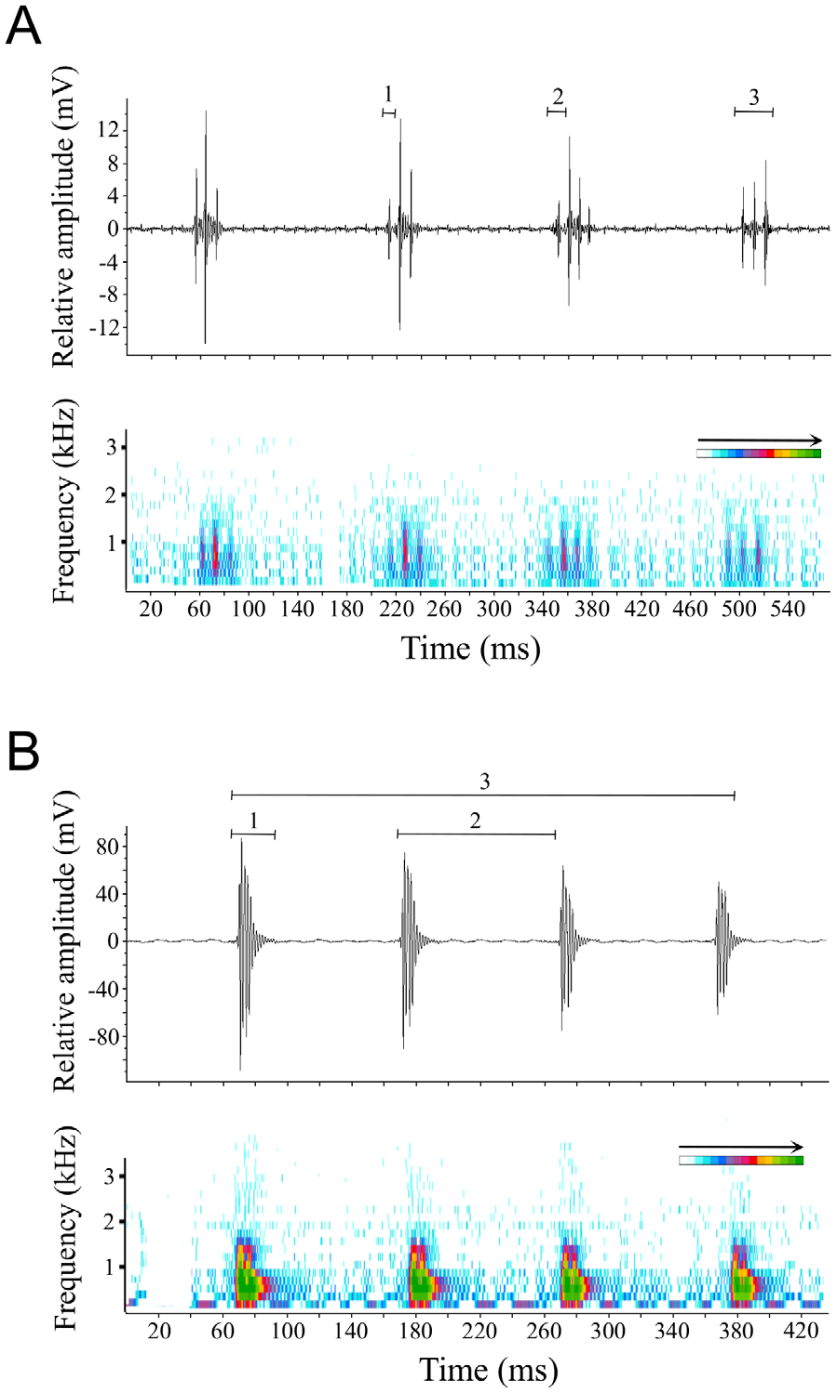
Example of agonistic sounds produced by *Amphiprion frenatus* during interactions. A) Oscillogram (top) and spectrogram (bottom) of submissive sounds produced by subordinate during head shaking movements. B) Oscillogram (top) and spectrogram (bottom) of aggressive sounds produced by dominant while displaying charge and chase. Note the differences in (1) pulse duration and (2) pulse period. The acoustic variable measured in (3) represents the sound duration in the case of submissive sounds, and the train duration in the case of aggressive sounds. The colour scale corresponds to the intensity associated with the different frequencies.

Submissive sounds were always associated with head shaking movements (Fig. 1), but fish could sometimes carry out these movements without vocalizing. Submissive sounds (*N* = 285 sounds analyzed for all individuals of the different groups; see Table 2) were produced when subordinates displayed submissive posture as a reaction to charge and chase by dominants, which means that these sounds were never recorded for the dominant females (rank 1) during this study. Generally speaking, submissive sounds are completely different from aggressive ones. They are always composed of several pulses whereas aggressive sounds are composed of a single pulse unit that can be emitted alone or in series (Fig. 2). They also exhibit shorter pulse periods and shorter pulse durations than aggressive sounds. In *A. frenatus*, submissive sounds can be produced alone or in series (2–9 sounds, 3.0±0.56), and are multiple-pulsed (2–6 pulses, 3.2±0.26). Pulse period averaged 11.8±2.4 ms and pulse duration ranged from 4.7 to 10.3 ms (7.9±2.15 ms). Sound period averaged 197.0±26.6 ms and sound duration ranged from 23.5 to 50.6 ms (35.9±9.59 ms). Pulses had peak frequency of 591±115 Hz and most sound energy ranged from 454 to 778 Hz.

**Table 2:** Summary (mean ± S.D.) of the seven acoustic features analyzed from submissive sounds produced by 9 *Amphiprion frenatus*.

Individual (*N*)	Pulse duration (ms)		Dominant frequency (ms)		Pulse period (ms)		Number of pulses per sound		Sound duration (ms)		Sound period (ms)		Number of sounds per train	
	Mean ± S.D.	*n*	Mean ± S.D.	*n*	Mean ± S.D.	*n*	Mean ± S.D.	*n*	Mean ± S.D.	*n*	Mean ± S.D.	*n*	Mean ± S.D.	*n*
Rank 2 (3)	9.9±0.9	300	482±54	300	14.3±1.8	91	3.3±0.9	20	46.6±11.9	91	216.2±43.8	56	2.7±0.7	33
Rank 3 (3)	8.7±0.9	300	559±60	300	11.8±1.4	99	3.0±0.7	20	36.6±9.7	98	192.5±33.5	71	3.1±1.3	34
Rank 4 (3)	5.2±0.6	300	731±100	300	9.2±0.9	96	3.2±0.8	21	25.8±7.3	96	172.3±31.3	73	3.7±1.3	27

*N*, number of individuals; *n*, number of data analyzed.

**Figure 3:**
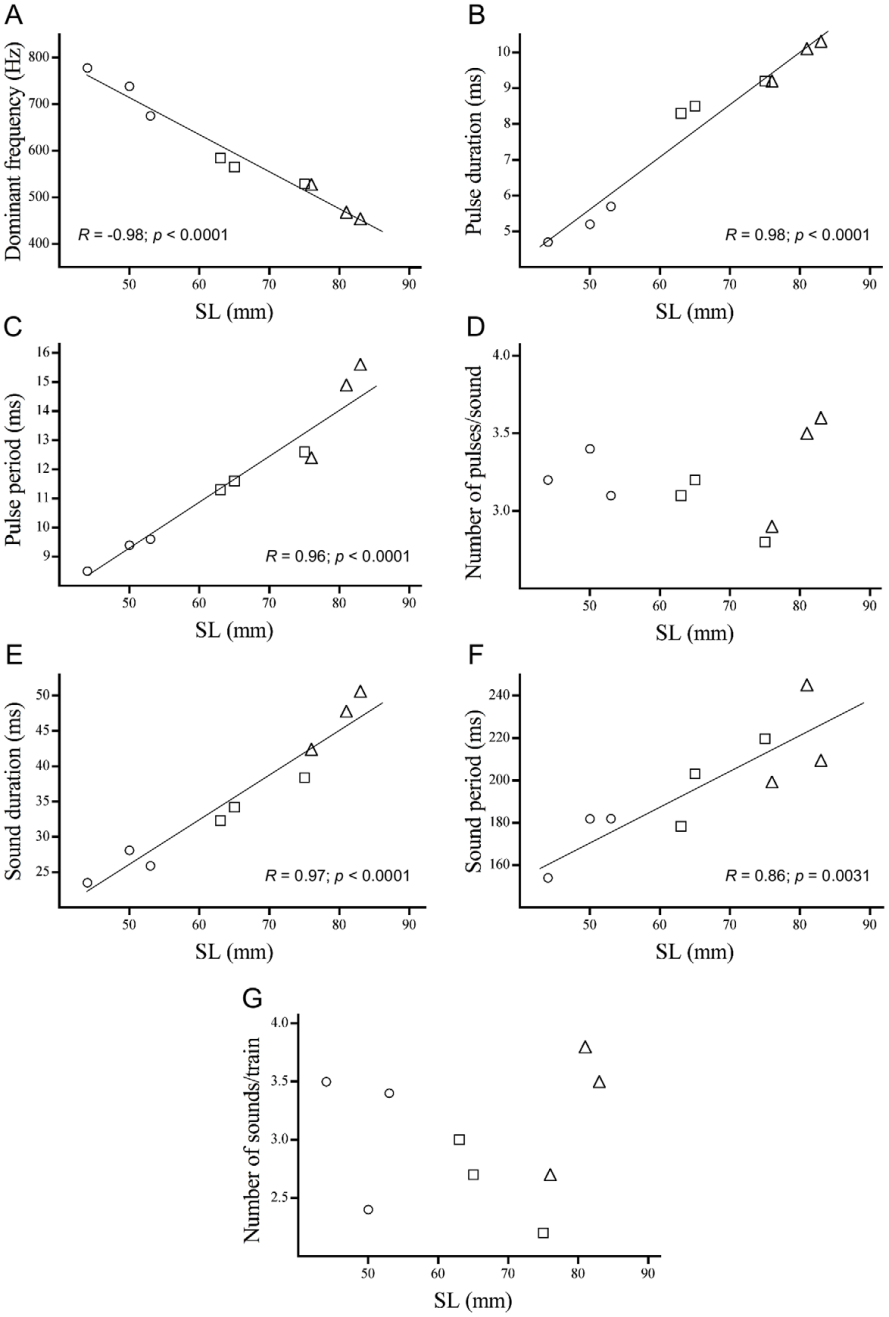
Influence of fish size (SL) on acoustic features of submissive sounds in *Amphiprion frenatus*. Correlations of (A) dominant frequency, (B) pulse duration, (C) pulse period, (D) number of pulses per sound, (E) sound duration, (F) sound period and (G) number of sounds per train against SL. Fish ranged from 44 to 112 mm in SL (*N* = 9). Results are expressed as mean values of all recorded pulses for each individual (○ = rank 4, □ = rank 3, ▵ = rank 2).

Dominant frequency and pulse duration were highly correlated with SL. Dominant frequency significantly decreased (*R* = −0.98, *p*<0.0001; Fig. 3A) whereas pulse duration significantly increased (*R* = 0.98, *p*<0.0001; Fig. 3B) with increasing SL. Pulse period was correlated across SL (*R* = 0.96, *p*<0.0001; Fig. 3C) and this sonic variable was also significantly correlated with pulse duration (*R* = 0.95, *p* = 0.0001). Additionally, sound duration was correlated with increasing SL (*R* = 0.97, *p*<0.0001; Fig. 3E); this acoustic feature being also significantly correlated with both pulse duration (*R* = 0.93, *p* = 0.0003) and pulse period (*r* = 0.98, *p*<0.0001). Likewise, sound period was correlated with increasing SL (*R* = 0.86, *p* = 0.0031; Fig. 3F), being significantly correlated with sound duration (*R* = 0.82, *p* = 0.0063). The number of pulses per sound did not change significantly (*R* = 0.10, *p* = 0.7917; Fig. 3D) across SL, as well as the number of sounds per train (*R* = 0.06, *p* = 0.8686; Fig. 3G).

A comparison of social rank values using SL as a covariate showed that the dominant frequency (ANCOVA, test for common slopes: F_(2,3)_ = 3.677, *p* = 0.156; test for intercepts: F_(2,5)_ = 1.204, *p* = 0.374) and pulse duration (ANCOVA, test for common slopes: F_(2,3)_ = 3.644, *p* = 0.175; test for intercepts: F_(2,5)_ = 4.204, *p* = 0.137) did not differ between individuals of different social ranks. Thereby, differences between social ranks in these acoustic features exclusively resulted from size differences. In addition, the influence of fish size on acoustic features was enhanced by comparing them between individuals of the same social rank but from different groups. All the acoustic features were significantly different between individuals of the same rank (Table 3), except when these ones had similar SL. In this case, acoustic features did not differ (Dunn's test, *p*>0.05).

**Table 3:** Comparison of the acoustic features of submissive sounds between individuals of the same social rank but from different groups of *Amphiprion frenatus*.

Acoustic variables	Social rank	H	*p*-value
Dominant frequency (Hz)	2	108.4	<0.0001
	3	42.11	<0.0001
	4	49.54	<0.0001
Pulse duration (ms)	2	79.16	<0.0001
	3	37.72	<0.0001
	4	139.0	<0.0001
Pulse period (ms)	2	121.5	<0.0001
	3	35.13	<0.0001
	4	64.19	<0.0001
Sound duration (ms)	2	6.418	0.0404
	3	15.18	0.0005
	4	10.27	0.0059
Sound period (ms)	2	9.995	0.0068
	3	22.80	<0.0001
	4	12.31	0.0021

H values are the result of the Kruskal-Wallis test (*df* = 2, *n* = 300). *n*, number of pulses analyzed.

Kruskal–Wallis one-way ANOVA revealed that means were significantly different between social ranks for pulse period (H = 6.489, *df* = 2, *p* = 0.0390) and sound duration (H = 7.200, *df* = 2, *p* = 0.0273), but not for sound period (H = 3.822, *df* = 2, *p* = 0.1479), number of pulses per sound (H = 1.898, *df* = 2, *p* = 0.3871) and number of sounds per train (H = 2.508, *df* = 2, *p* = 0.2853).

Pairwise comparisons showed that pulse period and sound duration were higher in rank 2 (Dunn's test, *p*<0.05; Table 2) than in rank 4, whereas no significant differences were observed between ranks 2 and 3 (Dunn's test, *p*>0.05; Table 2), and between ranks 3 and 4 (Dunn's test, *p*>0.05; Table 2) due to considerable overlap.

Aggressive and submissive sounds presented some acoustic features that displayed C.V._w_≤0.10 (Tables 4, 5), suggesting a strong homogeneity of these variables. All the acoustic features analyzed had C.V._b_/C.V._w_ ratios>1, showing a higher variability among than within individuals. Consistently, the Kruskal-Wallis analyses revealed significant differences among individuals for almost all features (Tables 4, 5), indicating that these acoustic variables (except the number of pulses per sound and the number of sounds per train in groups 1 and 2 of *A. frenatus*; Table 5) can potentially provide recognition cues to identify the sound emitter. The larger relative between-individuals variability (larger C.V._b_/C.V._w_ ratios) corresponded to the dominant frequency, pulse duration and pulse period (Tables 4, 5).

**Table 4:** Means, ± S.D., range, within-individuals variability (C.V._w_) and between-individuals variability (C.V._b_) for the four acoustic features analyzed from aggressive sounds produced by 14 *Amphiprion akallopisos*.

Acoustic variables	Overall Mean ± S.D. (range)	C.V._w_ (Mean)	C.V._w_ (range)	C.V._b_	C.V._b_/C.V._w_	H*	*p*-value
Dominant frequency (Hz)	663±199 (346–1207)	0.10	0.07–0.21	0.30	2.73	1577	<0.001
Pulse duration (ms)	13.4±3.9 (3.8–22.9)	0.10	0.06–0.18	0.29	2.90	1614	<0.001
Pulse period (ms)	75.9±13.4 (32.4–121.9)	0.15	0.09–0.21	0.23	1.53	203.9	<0.001
Number of pulses per sound	3.9±2.2 (2–14)	0.50	0.30–0.68	0.56	1.12	25.55	0.0195

* Results of Kruskall-Wallis test (*df* = 13, *n* = 1818) comparing differences between 14 individuals of *A. akallopisos* for each acoustic feature. Note that these values were calculated based on acoustic data obtained from Colleye et al. (2009). *n*, number of pulses analyzed.

**Table 5:** Means, ± S.D., range, within-individuals variability (C.V._w_) and between-individuals variability (C.V._b_) for the seven acoustic features analyzed from submissive sounds produced by 9 *Amphiprion frenatus*.

Acoustic variables	Group number	Overall Mean ± S.D. (range)	C.V._w_ (Mean)	C.V._w_ (range)	C.V._b_	C.V._b_/C.V._w_	H*	*p*-value
Dominant frequency (Hz)	1	631±135 (431–1036)	0.10	0.09–0.15	0.21	1.90	213.7	<0.001
	2	593±139 (431–862)	0.10	0.08–0.12	0.22	2.20	251.0	<0.001
	3	552±105 (345–776)	0.09	0.08–0.11	0.19	2.11	239.9	<0.001
Pulse duration (ms)	1	7.4±2.1 (3.8–11.2)	0.10	0.09–0.11	0.28	2.80	232.4	<0.001
	2	8.5±2.8 (3.6–15.1)	0.10	0.09–0.11	0.34	3.40	250.8	<0.001
	3	9.1±2.8 (4.5–13.9)	0.08	0.06–0.12	0.31	3.87	233.2	<0.001
Pulse period (ms)	1	10.7±2.0 (6.7–15.4)	0.10	0.08–0.12	0.19	1.90	143.8	<0.001
	2	11.9±2.5 (7.4–17.6)	0.10	0.08–0.11	0.21	2.10	167.4	<0.001
	3	12.7±2.6 (8.2–18.4)	0.08	0.05–0.09	0.21	2.62	176.2	<0.001
Number of pulses per sound	1	3.1±0.6 (2–5)	0.20	0.19–0.23	0.21	1.05	5.36	ns
	2	3.4±0.9 (2–6)	0.26	0.21–0.31	0.27	1.04	2.59	ns
	3	3.1±0.8 (2–5)	0.25	0.22–0.29	0.27	1.08	12.86	<0.01
Sound duration (ms)	1	29.2±8.4 (9.8–48.2)	0.22	0.18–0.24	0.29	1.32	59.24	<0.001
	2	36.9±14.1 (16.6–72.4)	0.28	0.22–0.33	0.38	1.36	25.94	<0.001
	3	36.6±14.1 (15.0–73.8)	0.24	0.13–0.35	0.38	1.58	49.90	<0.001
Sound period (ms)	1	176.4±36.9 (92.2–295.3)	0.18	0.16–0.21	0.21	1.17	16.53	<0.01
	2	206.3±40.9 (135.6–280.9)	0.15	0.12–0.18	0.20	1.33	21.07	<0.001
	3	201.7±31.5 (118.3–266.3)	0.13	0.08–0.16	0.16	1.23	16.17	<0.01
Number of sounds per train	1	3.3±1.5 (2–9)	0.39	0.28–0.53	0.44	1.13	4.36	ns
	2	3.2±1.3 (2–7)	0.35	0.30–0.41	0.41	1.17	4.93	ns
	3	2.6±0.8 (2–5)	0.23	0.18–0.27	0.30	1.30	14.39	<0.01

* Results of Kruskal-Wallis test (*df* = 2, *n* = 300) comparing differences between individuals in a group of *A. frenatus* for each acoustic feature. *n*, number of pulses analyzed.

### Reproductive sounds

A total of eight spawning events were observed. All reproductive patterns including nest preparation, courtship, spawning and parental care were once observed and recorded in *A. akindynos*, *A. melanopus* and *A. percula* during July 2008 at Oceanopolis Aquarium. *Amphiprion clarkii* spawned four times between May and July 2009 in Sesoko Station, and all the reproductive activities were observed and recorded. Spawning always occurred in the afternoon from 2:00 to 5:00 p.m., whatever the species. In addition, one complete spawning sequence in *A. perideraion* was observed and recorded in the field for approximately 80 minutes in August 2009 (11:20 to 12:40 a.m.).

Overall, the most striking observation was the complete absence of sound production throughout all activities of the reproductive period in the different clownfish species recorded, and whatever the recording environment (aquarium or field). However, some other typical behaviors seem to be responsible for the synchronization of the reproductive activities.

1. The arrival of spawning period was indicated by an increase of cleaning activity by the male, and by the belly of the female that was noticeably distended (especially in *A. percula* and *A. perideraion*). These features became usually distinct three or four days before spawning. Occasionally, fish chased each another or engaged in fast side-by-side swimming and belly touching; the female being the initiator in most of these encounters. Sometimes, the female entered the nest and pressed her belly against the rock (spawning ground). Pecking movement of the male at the surface of nest became more vigorous from about two hours before spawning; this movement was continued until just before spawning.

About 15 minutes before spawning, nest-cleaning activities was more rigorously carried out by the female, which pecked the surface of the spawning ground. Also, she pressed her belly against the substrate. These activities seemed to aim at making sure of the completion of the spawning ground. At the onset of spawning, the whitish cone-shaped ovipositor of the female was clearly apparent.

2. The spawning was carried out in the following way: the female entered the nest, pressed her belly against the spawning ground and swam slowly in a circular path; the male followed closely behind and fertilized the spawn. Locomotion during the spawning passes was achieved by rapid fluttering of the pectoral fins. The male frequently mouthed the eggs during the spawning period. Both fish also nibbled on the tips of the anemone tentacles for preventing the spawn from entering into contact with them.

3. After the spawning, the incubation period took place and was characterized by parental care, which lasted usually six to seven days. The male assumed nearly the full responsibility of tending the nest. Except an initial moderate level of activity at spawning, there were no cleaning activities the first two days. Then, an abrupt increase occurred the next few days until hatching. Two basic nest-caring behavior patterns were observed. Fanning was the most common and was mainly performed by fluttering the pectoral fins. Mouthing the eggs and substrate biting at the periphery of the nest were also exhibited. Dead eggs were regularly removed as indicated by bare patches at the nesting surface.

## Discussion

In clownfishes, different types of sounds such as “threatening” and “shaking” [18], “click” and “grunt” [16] or “pop” and “chirp” [19], [23] have already been described during interactions between conspecifics. Although a dichotomy in sounds was reported in each case, these terms have been inconsistently applied and were not always supported by appropriate data which create confusion [10]. Consequently, it remained difficult to match a type of sound with a given behavior. However, our multiple observations highlight that submissive sounds (i.e. chirps, see [23]) are clearly different from aggressive sounds (i.e. pops, see [23]). Aggressive sounds are mainly produced by dominants during chases and threat displays between conspecifics [20], whereas submissive sounds are always emitted when subordinates exhibit head shaking movements in reaction to aggressive displays by higher-ranking individuals. Therefore, both types of sounds seem to be an integral part of the agonistic behavior in clownfishes. Given that they present some differences in sound spectra and shape of the temporal envelope, it is important to emphasize that these two types of sounds would result from two different mechanisms. Aggressive sounds result from jaw teeth snapping [21], [28] but the sound-producing mechanism of submissive sounds is still unknown.

### Importance of size-related acoustic signals for the group hierarchy

**Figure 4:**
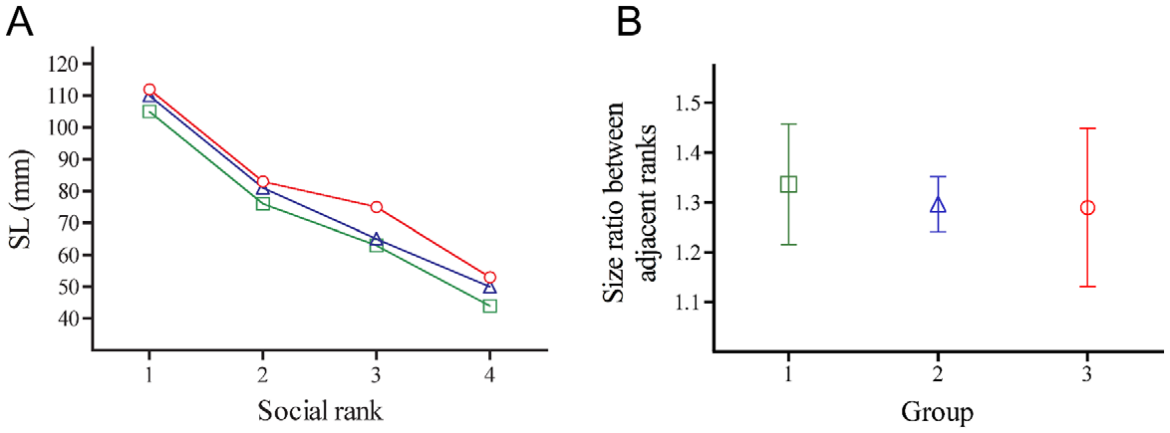
Fish size (SL) and size ratios of individuals adjacent in rank within each group of *Amphiprion frenatus*. A) The observed distribution of fish size (SL) within each group. B) Distribution of body size ratios between individuals adjacent in rank within each group. Results are expressed as mean ± S.D. values (□ = group 1, ▵ = group 2, ○ = group 3).

Interestingly, dominant frequency and pulse duration of submissive sounds display size-related variation in some acoustic features. The more fish size increases, the more dominant frequency decreases, and the more pulse duration increases. The same relationships have already been found for aggressive sounds among 14 different species [21]. Differences in both sound characteristics were related to fish size and not to sexual status or social rank. However, size, sex and social rank are extremely related to each other due to the size-based hierarchy within each group [14], [15]. In *A. percula*, Buston and Cant [29] demonstrated that individuals adjacent in rank are separated by body size ratios whose distribution is significantly different from the distribution expected under a null model: the growth of individuals is regulated such that each dominant ends up being about 1.26 times the size of its immediate subordinate. The same kind of ratio (≈1.30) is observed in the different groups of *A. frenatus* of this study (Fig. 4). The respect of this ratio within groups highlights that dominant frequency and pulse duration can be signals conveying information on the social rank of the emitter within the group.

Aggressive and submissive sounds are involved in interactions between group members (Video S1, S2). Being associated with a specific display, they might have a different function within the group. Indeed, aggressive sounds could possess a deterrent function by giving a reminder signal of dominance during interactions whereas submissive sounds could possess an appeasement function by expressing the lower rank status during interactions. Likewise, two different types of sounds are emitted by the grey gurnard *Eutrigla gurnardus* depending on the interactions between individuals: knocks are produced during low levels of aggression and grunts mainly while performing frontal displays to opponents [30].

Clear differences were found among aggressive and submissive sounds attributed to different individuals. All acoustic variables were significantly more variable between than within individuals and thus could all potentially provide cues to identify individuals. Furthermore, the most important variables to allow individual identification were dominant frequency and pulse duration for both types of sounds (Tables 4, 5). Pulse period, in a lesser extent, was also consistently important for discriminating among individuals in the case of submissive sounds (Table 5). In order to be good candidates for individual recognition, these acoustic features should propagate through the environment, and should be detected by the receiver. Sound propagation in shallow water can result in signal degradation over short distances, including sound pressure level and frequency attenuation, and sound duration loss [31]. However, the effect of environmental attenuation and signal degradation should not impose a major restriction within a group of clownfishes since all individuals inhabit a restricted territory (the sea anemone) and spend most of the time in close vicinity of their host.

Since dominant frequency and pulse duration of both aggressive and submissive sounds are size-dependent, temporal and spectral inter-individual differences might be detected by members within the group. Teleost fishes such as *Gobius niger* (Gobiidae) and *Sparus annularis* (Sparidae) are able to discriminate tonal sounds differing in frequency of approximately 10%; the frequency discrimination ability at 400 Hz is approximately 40 Hz [32]. In the clownfish *A. akallopisos*, aggressive sounds emitted by non-breeders, males and females differ in dominant frequency by >10% [20]. In *A. frenatus*, sounds emitted by individuals of different social ranks also differ in dominant frequencies by >10% (Table 2). Such an ability to discriminate frequency differences has already been observed in some pomacentrids. In the damselfish *Abudefduf saxatilis*, fish size has a significant effect on auditory sensitivity [33]: all fish are most sensitive to the lower frequencies (100–400 Hz) but the larger ones are more likely to respond to higher frequencies (1000–1600 Hz). The effect of fish size on hearing abilities was also supposed in three different clownfish species [34]. Although the best hearing sensitivity is around 100 Hz, small individuals were more sensitive to a larger frequency interval (100–450 Hz), and thus they are more sensitive to the frequencies emitted by larger conspecifics.

No information related to fish size can be extracted from number of pulses per sound or number of sounds per train. Differences in these acoustic features appear to be related to a difference in motivation. Motivation is known for playing a role in damselfishes, regarding their sounds produced during aggression. In *Dascyllus albisella* and *D. flavicaudus*, aggressive sounds are different according to whether they are emitted towards conspecifics or heterospecifics, being multiple-pulsed or single-pulsed, respectively [12], [13]. In *Pomacentrus partitus*, the frequency of sounds by a territorial resident is relatively low at the territorial border, but it rapidly increases as intruder approaches the residence [35]. In the clownfish *A. akallopisos*, the most aggressive males were characterized by a higher number of pulses per sound and a shorter pulse period (pers. obs.). The smallest individuals (rank 4) in *A. frenatus* groups emitted the highest number of sounds per train (Table 2). All these variations in acoustic features might be related to the willingness to express the position within the group hierarchy. For example, lower-ranking individuals might produce more submissive sounds in order to limit aggressive acts from dominants.

### No reproduction-related sound

Unlike observations made by Takemura [22], no acoustical behavior was observed during reproductive activities in clownfishes. Moreover, Takemura's data are somewhat doubtful since *A. ocellaris*, *A. frenatus* and *A. sandaracinos* would emit sounds with high frequency component of more than 2 kHz during reproduction [22]. According to hearing sensitivity in clownfishes (*A. frenatus*, *A. ocellaris* and *A. clarkii*), the frequency range over which they can detect sounds is between 75 and 1800 Hz, and they are the most sensitive to frequencies below 200 Hz [34]. Therefore, this finding raises the question over the interest of clownfishes to produce sounds they could not detect during reproductive activities. It remains these sounds could just be a by-product of the nest cleaning activities.

In the field, clownfishes spawn on average from 1.0±0.5 to 0.6±0.1 times per month depending on whether they live in tropical waters [16], [36] or in more temperate regions [37], [38], [39]. In captivity, the spawning frequency is higher and on average 25±5.3 times per year [40], [41]. This frequency was observed at Oceanopolis Aquarium with captive clownfishes for which spawning occurred approximately every two weeks. In this context, it could be argued that the captivity modulates some aspects of the behavior [40] such as sound production during reproduction. However, 1) the pair of *A. clarkii* reared at Sesoko Station showed the same spawning frequency (∼every 2 weeks), although its reproduction was restricted to summer season and it was reared under semi-natural conditions (i.e. outdoor tank filled with running seawater and maintained under natural photoperiod); 2) spawning was witnessed in the field for *A. perideraion*, and no sound was produced by the mating pair during the reproductive event. Yet, recording of aggressive sounds during the same session supports the fact that the recording material worked well.

Overall, sound production does not seem to be involved in the reproductive behavior of clownfishes, which might be explained by some particular aspects related to their way of life.

The reproductive behavior of pomacentrids is subdivided into the following major categories [42], [43]: 1) establishment of territory, 2) selection of nest site within the territory, 3) preparation of the nest site, 4) courtship and pair formation, 5) spawning and fertilization, and 6) parental care. Clownfishes conform to this general pattern but are distinctive with regards to formation of permanent pair bonds that usually last for several years in most species [16], [44]. In other damselfishes, one male may mate with several females during a single spawning episode [42], [44]. In clownfishes, male does not need to exhibit typical courtship behavior for attracting female. Pair-bonding is very strong and is correlated by the small size of their territories (centered on actinians) that is, in turn, correlated with the unusual social hierarchy existing in each social group. On the other hand, it seems that other cues such as visual signals might be useful for synchronizing reproductive activities. Just before spawning occurs, the female joins the male and becomes more insistent in the nest-cleaning activities, probably in order to convey visual cues about its readiness to spawn. Likewise, it is possible that the male regulates its level of nest-caring activity in response to visual stimuli received when inspecting eggs [16]. A visual stimulus of this sort would signal the stage of egg development and the need for increased fanning and mouthing activities. Allen [16] experimentally demonstrated that strong agitation of the eggs is a requisite for hatching. He also noted that there was a pronounced increase in the amount of male nest care on day six of incubation. On that day, the embryos are well developed with one of the most noticeable features being the large eyes with their silvery pupils. Such a feature might serve as an appropriate visual cue. Therefore, other cues such as visual and perhaps chemical signals might be involved in reproductive activities. However, new behavioral tests would need to be run to determine the proper role of such signals during the reproduction of clownfishes.

## Conclusion

Unlike other pomacentrids, sounds are not produced for mate attraction in clownfishes. It is likely an evolutionary outcome related to their peculiar way of life: these fishes form small social groups including only one mating pair, inhabit a restricted territory (the sea anemone), spend most of the time in close vicinity of their host and rarely interact with other species on the reef. On the other hand, sounds seem to be important to reach and to defend the competition for breeding status. Although they are restricted to agonistic interactions only, acoustic signals seem to be an integral part of their daily behaviors. The implication of acoustic signals in agonistic interactions may be an interesting strategy with an economic way for preventing conflicts which otherwise might escalate to a severe outcome.

Clownfish sounds can be divided into two main categories: aggressive sounds produce in conjunction with threat postures (charge and chase), and submissive sounds always emit when subordinates exhibit head shaking movements in reaction to aggressive displays by dominants. Both types of sounds show intraspecific differences related to fish size, highlighting that some acoustic features (i.e. dominant frequency and pulse duration) might be useful cues for individual recognition within the group. These observations are of significant importance because the social structure of clownfishes strictly relies on a size-based dominance hierarchy.

## Supporting Information

Video S1 **Implication of aggressive sounds during agonistic interactions between group members in the field.** Note that a fish is chasing another one (smaller) while producing a series of aggressive sounds.
(AVI)

Video S2 **Behavioral posture (head shaking movements) exhibited by subordinates while producing submissive sounds.** Note that fish make sounds while doing lateral quivering of the body that begins at the head.
(AVI)
